# Treatment costs and cost drivers among osteoporotic fracture patients in Japan: a retrospective database analysis

**DOI:** 10.1007/s11657-018-0456-2

**Published:** 2018-04-25

**Authors:** Yurie Taguchi, Yuta Inoue, Taichi Kido, Nobuhiro Arai

**Affiliations:** 1 Amgen Astellas BioPharma K.K, 1-7-12 Marunouchi, Chiyoda-ku, Tokyo, Japan; 2 Deloitte Tohmatsu Consulting LLC, 3-3-1 Marunouchi, Chiyoda-ku, Tokyo, Japan

**Keywords:** Osteoporosis, Fracture, Cost, Japan, Comorbidity, Database

## Abstract

**Summary:**

This study estimated the direct medical costs of osteoporotic fractures from a large claim database in Japan. We further identified several comorbidities which drove the treatment costs. The results would contribute to health economic analysis as well as understanding of individual financial burden in Japan.

**Introduction:**

The purposes of this study were to estimate treatment costs of osteoporotic fractures and to investigate the cost drivers.

**Methods:**

Male and female patients aged 50 years and older with a hip, vertebral, or non-hip/non-vert (NHNV) fracture between April 2008 and December 2016 were analyzed from claim database. Two types of costs were estimated. The incremental yearly costs of fractures and comorbidity treatments (total medical costs) were calculated by subtracting pre-fracture costs from post-fracture costs. The costs exclusive for fracture treatments (fracture treatment costs) were estimated by summing up the costs of fracture treatments within 1 year after fracture. The associations between comorbidities and costs were examined with a generalized linear model.

**Results:**

Total 12,898 patients were identified (83% was female). The total medical costs of fractures were $14,592 for male-hip, $15,691 for female-hip, $4268 for male-vertebral, $3819 for female-vertebral, $3790 for male-NHNV, and $4259 for female-NHNV. The fracture treatment costs were $4506 for male-hip, $5427 for female-hip, $1022 for male-vertebral, $1044 for female-vertebral, $1035 for male-NHNV, and $1408 for female NHNV. Three comorbidities were associated with increasing fracture treatment costs whereas four comorbidities were associated with decreasing fracture treatment costs. Five comorbidities were associated with increasing total medical costs whereas one comorbidity was associated with decreasing total medical costs.

**Conclusions:**

Yearly treatment costs were increased considerably after fracture. Several comorbidities were considered to be cost drivers for osteoporotic fracture treatment. The cost estimates with different patient profile would support conducting health economic analysis in the future.

## Introduction

Osteoporotic fractures often have serious health impacts. For example, in a previous research, those with osteoporotic fractures had a higher mortality rate and decreased individual quality of life [1, 2]. It is established that a risk of osteoporosis and osteoporotic fracture increases as age increases [3], and Japanese population is going through the rapidly increasing elderly population. In 2015, people aged 65 years and older consisted 26.6% of total population, and it was estimated that this proportion would increase to 37.7% by 2050 [4]. Furthermore, particularly in Japan, the financial burden of osteoporotic fracture is immense. Moriwaki and Kamae estimated that the financial burden due to osteoporotic hip and vertebral fracture was 27.5 billion JPY (250 million USD when 1 USD = 110 JPY) in 2009 [5].

Under this situation, the health economic analysis in osteoporosis and osteoporotic fracture is featured more than ever. In recent years, new drugs, such as teriparatide and denosumab, have been launched in Japan with higher drug prices than conventional bisphosphonates. In line with the increasing varieties of osteoporosis drugs, attention by researchers has been paid to cost-effectiveness of these drugs. Ding et al. (2008) analyzed the cost-effectiveness of risedronate sodium compared with no-treatment [6]. Moriwaki and his colleagues examined cost-effectiveness of alendronate versus no-treatment in 2013 as well as cost-effectiveness of zoledronic acid versus alendronate in 2017 [7, 8]. Mori et al. (2017) reported cost-effectiveness of denosumab versus alendronate in 2017 [9]. In terms of treatment cost parameters such as the yearly treatment costs for fractures, most studies used the cost input retrieved from the Japanese medical fee schedule with a set of treatment assumptions or estimates based on small-sized patient population. This is because there has been no study which estimated the treatment costs of osteoporosis and osteoporotic fractures with large-sized real-world data (RWD). Given that a considerable size of claim database has recently become commercially available, estimating osteoporotic fracture treatment costs using them is considered valuable.

Therefore, the primary objective of this study is to estimate the current yearly treatment costs due to osteoporotic fractures as well as treatment costs of osteoporosis patients in Japan using large-sized RWD. It will not only help estimate the financial burden of osteoporotic fracture but also help conduct various health economic analyses. The secondary objective of this study is to identify cost drivers among osteoporotic fracture patients. We defined cost drivers as the factors associated with increased or decreased treatment costs of osteoporotic fracture hereafter and explored them via the retrospective database analysis.

## Methods

### Data source

In this retrospective observational study, the medical claim database provided by Medical Data Vision Co. Ltd. (MDV; Tokyo, Japan) was used. MDV accumulated anonymous patient-level medical claim data from more than 17.23 million unique patients in more than 240 acute-care hospitals, which is almost one-eighth of the total Japanese population [10].

The MDV database includes patient demographics (age and gender), resource use, and diagnosis history including the date when it was first diagnosed at enrolled hospitals. The resource use history covers amounts used and prices for each resource (drug/medical procedure/tools). However, it does not contain socio-economic status (SES) records, patient behavioral records such as smoking history for outpatients, and treatment outcome records (i.e., cured/dead/transferred) in outpatient service.

### Study population

Patients aged 50 years and older with diagnosis of osteoporotic fracture, dating from April 2008 to January 2017, were identified from the database using International Classification of Diseases (10th revision) (ICD-10). Patients with osteoporotic fractures were defined as those with M80 (osteoporosis with pathological fracture) or those with both M81 (osteoporosis without fracture) and ICD-10 code for fractures which can be assumed as osteoporotic fractures. These fractures were decided based on the guidelines and expert opinion, and categorized into three groups: hip, vertebral, and non-hip and non-vertebral (NHNV) fractures [3]. The list of fractures is as follows: hip (S72.0, S72.1, S72.2, and S32.4); vertebral (S12, S22, and S32 excluding S32.3-5); and NHNV (S22.3-5, S32.3, S32.5, S42.2, S42.4, S52.0-1, S52.3, S52.5-6, S72.3-4, S72.7, S72.9, S82.0-1, and S92.0).

The index date was defined as the date when the fracture was first diagnosed since they were enrolled in the database. If a patient develops a fracture of different ICD-10 code from the first one within 1 year, the fracture was defined as the second fracture. Patients who had a record on January 2017 were excluded as they may still continue their treatments thereafter. As a result, we used the patient data from April 2008 to December 2016. In addition, patients whose initial records or final records in the database were within 1 year from the index fracture were excluded to avoid including those who changed hospitals or died within 1 year.

### Treatment costs

Two types of treatment costs were estimated in this study. One is the cost incurred by any medical resource use due to fracture development, and this was named as “total medical cost.” The other is the cost incurred by the medical resource use for osteoporosis and fracture treatments, and this was named as “fracture treatment cost.” Medical costs in Japan can be categorized into four: doctor consultation costs, drug costs, non-drug treatment costs (including diagnosis), and inpatient specific costs (such as room charges). The “total medical costs” includes all of these whereas the “fracture treatment cost” includes drugs and non-drug treatment costs related to fracture and general orthopedic treatments. The total medical cost was estimated by subtracting the treatment costs within 1 year before index fracture from the treatment costs within 1 year after index fracture. The fracture treatment cost was estimated by aggregating the costs for the specific medical resources used for fracture treatments within 1 year after fracture development. These resources were defined by using two reference books which describe the resources by disease indication [11, 12]. The resources with indication of fracture and general orthopedic treatments were extracted from these books. When estimating yearly costs for the second fracture, 1 year since the date of second fracture development was defined as a period for estimation.

Costs were calculated by multiplying the amount used by the unit price for each resource use record from public health care payer’s perspective. The prices used in this study were adjusted to the latest unit prices issued in 2017 by the government, which are applied to every hospital/clinics in Japan. All the costs were transformed into USD (assuming 1 USD = 110 JPY).

### Data analysis

Both the total medical costs and the fracture treatment costs were estimated at patient level and reported as means with 95% confidence interval (CI). Before reporting the descriptive statistics, data with extreme values in the total medical costs were excluded as outliers. The data were stratified by gender and fracture category, then a box-and-whisker plot was made for each subgroup. The outliers were defined as those outside the whisker. When comparing the costs of those patients with the second fracture to that of single fracture patients, *t* test was used for the total medical costs and Wilcoxon ranked sum test was used for the fracture treatment costs, as it was expected that the fracture treatment costs were skewed compared with the total medical costs.

Comorbidity profiles of the patients were also gathered to use as explanatory variables. Both highly frequent comorbidities and diseases associated with osteoporosis risk were included. To extract highly frequent comorbidities, prevalence rates of comorbidity within 3 months before and after index fracture were calculated for each of three cost groups (A: top 30% tiles; B: middle 40% tiles; and C: bottom 30% tiles according to the total medical costs). The following two criterion were used to determine the comorbidities included in the later analysis: (1) If prevalence rates were more than 30% for all the three groups or (2) a prevalence rate in the group A was more than 20%. Medical conditions with M45–49 in ICD-10 code (spondylopathies) were excluded from the list, as it was assumed to be highly correlated with vertebral fractures. To extract the comorbidities associated with osteoporosis risk, those described as major risk factors in the guidelines for prevention and treatment of osteoporosis were also included.

To identify the cost drivers for fracture treatment costs based on patient profiles (demographic factors, fracture type, treatment period reflecting both inpatient stay and outpatient visit, and comorbidities), a generalized linear model (GLM) with gamma family with log link function was applied for patients with single fracture. The increased or decreased association was further examined by conducting a subgroup analysis of the patients with one type of fracture (hip or vertebral). In this subgroup analysis, the driving factors for the total medical costs were also tested by a GLM with Gaussian family and identity link function. In this regression analysis with the total medical cost, the associations between patient profiles (demographic factors, fracture type, treatment period, treatment costs in the year before fracture, and comorbidities) and change in the total medical costs were examined. Drivers for the treatment costs of NHNV fracture were not examined because NHNV fractures consist of fractures at different body parts (e.g., radius/ulna or chest), and its result would be difficult to interpret as we would not be able to identify which NHNV fracture was driven by a particular factor. All statistical analyses were performed using R version 3.3.3 (R foundation for statistical computing, Vienna, Austria).

## Results

### Participant characteristics

From April 2008 to December 2016, a total of 14,001 patients were identified as patients with a single osteoporotic fracture for the analysis. Of those patients, the fracture treatment costs were incurred among 12,898 patients. These 12,898 patients were included for the later analysis because the remaining 1103 patients were assumed to be those who did not receive fracture treatments at the enrolled hospitals but may have received at different hospitals. While there was little difference in gender proportion, age group, and fracture type between these two datasets, total costs were generally higher among dataset of those 12,898 patients. Their characteristics are shown in Table 1. It was found that 10,755 patients (83%) were female patients, and the patients aged 70–89 consisted large proportion (male 80%, female 76%). Although the treatment costs incurred were more expensive in male than in female regardless of pre- or post-fracture, the total medical costs were higher in female than in male.

**Table 1:** Patient characteristics at index fracture

	Total population with single fracture and fracture treatment cost > 0				Multiple vertebral fracture patients	
	Male		Female		Female	
	*n* = 2143		*n* = 10,755		*n* = 100	
Age						
Mean (SD)	77.7	(8.4)	77.6	(8.7)	78.9	(8.6)
50–59, *n* (%)	73	(3%)	311	(3%)	3	(3%)
60–69, *n* (%)	261	(12%)	1589	(15%)	8	(8%)
70–79, *n* (%)	830	(39%)	4050	(38%)	36	(36%)
80–89, *n* (%)	877	(41%)	4038	(38%)	46	(46%)
90-, *n* (%)	102	(5%)	767	(7%)	7	(7%)
Fracture type, *n* (%)						
Hip	341	(16%)	2074	(19%)	–	
Vertebral	1407	(66%)	5304	(49%)	–	
NHNV	395	(18%)	3377	(31%)	–	
Treatment period, mean (SD)	9.1	(4.1)	9.3	(4.0)		
Medical cost (USD), mean (SD)						
1 year before (total fracture)	8200	(13,971)	4310	(7867)	–	
1 year after (total fracture)	14,023	(15,197)	10,557	(10,852)	–	
Total medical cost (difference)	5823	(8898)	6246	(8522)	–	
Fracture treatment cost (USD), mean (SD)						
1 year after (total fracture)	1579	(2577)	2004	(2968)	–	

Only female patients with the second vertebral fractures were reported in this study, since male patients with the second fractures and female patients with the second fractures that were not vertebral fractures were very few. For this reason, in order to analyze the impact of the second fractures on individual treatment costs, 100 female patients with the second vertebral fractures alone were included in the comparative analysis of fracture costs between patients with single fracture and those with the second fracture (in the next section). Their mean age was 78.9, which was slightly higher than the mean age of female patients with single vertebral fracture (77.6 [not reported in the table]).

### Cost profiles by fracture site

Treatment costs incurred were stratified by gender and fracture site as shown in Table 2. Significantly higher total medical costs were observed in hip fracture patients of both genders (male $14,592 [95% CI $13,356 to $15,829], and female $15,691 [95% CI $15,250 to $16,132]) compared to other two types of fractures. Cost estimates for vertebral and NHNV fractures were not largely different (male-vertebral: $4268 [95% CI $3891 to $4645]; female-vertebral: $3819 [95% CI $3661 to $3977]; male-NHNV: $3790 [95% CI $3094 to $4486]; female-NHNV: $4259 [95% CI, $4033 to $4484]).

**Table 2:** Cost profiles among analyzed population by fracture site

	Total population with fracture treatment cost > 0		Multiple vertebral fracture patients
	Male	Female	Female
	*n* = 2143 Mean (95% CI)	*n* = 10,755 Mean (95% CI)	*n* = 100 Mean (95% CI)
Total medical cost (USD)			
Hip	14,592 (13,356 to 15,829)	15,691 (15,250 to 16,132)	–
Vertebral	4268 (3891 to 4645)	3819 (3661 to 3977)	–
NHNV	3790 (3094 to 4486)	4259 (4033 to 4484)	–
Multiple vertebral	–	–	7651 (3939 to 11,364)
Fracture treatment cost (USD)			
Hip	4506 (4093 to 4920)	5427 (5253 to 5601)	–
Vertebral	1022 (929 to 1114)	1044 (996 to 1092)	–
NHNV	1035 (862 to 1208)	1408 (1340 to 1476)	–
Multiple vertebral	–	–	2458 (1763 to 3153)

Similar result was observed in the fracture treatment costs. The significantly higher costs were observed in hip (male $4506 [95%CI $4093 to $4920]; female $5427 [95%CI $5253 to $5601]) and no large difference was observed between the vertebral and NHNV fracture patients’ costs. Among hip fracture patients, the fracture treatment costs were higher in female.

When comparing the costs of the second vertebral fracture female patients with the costs of the single vertebral fracture female patients, the second fracture costs were significantly higher in both the total medical costs and the fracture treatment costs (for the total medical costs, mean was $7651 in the second vertebral fracture and $3819 in the single vertebral fracture; *p* < 0.05. For the fracture treatment costs, mean was $2458 in the second vertebral fracture and $1044 in the single vertebral fracture; *p* < 0.01).

### Comorbidity profiles for osteoporotic fracture patients

Comorbidity profiles of the studied population is shown in Table 3. Twelve comorbidities (GI diseases, hypertension, other functional intestinal disorder, dorsalgia, disorder of lipoprotein metabolism and other lipidemia, sleep disorders, diabetes, osteoarthritis of knee, angina pectoris, heart failure, disorder of refraction and accommodation, and iron deficiency anemia) met with the eligible criteria. The lists of comorbidities selected based on the criteria were not different between male and female.

**Table 3:** Comorbidity profiles among analyzed population

Comorbidity	ICD10	Total population with fracture treatment cost > 0			
		Male		Female	
		*n* = 2143 (%)		*n* = 10,755 (%)	
GI diseases^a^	K21,K25, K29	1498	(70%)	7165	(67%)
Hypertension	I10-I15	1200	(56%)	5567	(52%)
Other functional intestinal disorder	K59	1018	(47%)	4389	(41%)
Dorsalgia	M54	834	(39%)	3846	(36%)
Disorders of lipoprotein metabolism and other lipidemia	E78	585	(27%)	3649	(34%)
Sleep disorders	G47	649	(30%)	3406	(32%)
Diabetes	E10-E14	743	(35%)	2853	(27%)
Osteoarthritis of knee	M17	260	(12%)	2689	(25%)
Angina pectoris	I20	486	(23%)	1855	(17%)
Heart failure	I50	496	(23%)	1801	(17%)
Disorder of refraction and accommodation	H52	362	(17%)	1776	(17%)
Iron deficiency anemia	D50	359	(17%)	1554	(14%)
COPD	J40–44, J47	402	(19%)	1127	(10%)
Chronic kidney disease	N18	241	(11%)	396	(4%)
Thyrotoxicosis	E05	28	(1%)	172	(2%)
Hypofunction and other disorders of the pituitary gland	E23	9	(0.42%)	24	(0.22%)
Primary hyperparathyroidism	E210	2	(0.09%)	7	(0.07%)
Cushing’s syndrome	E24	0	(0%)	4	(0.04%)

^a^Gastrointestinal (GI) diseases includes gastro-esophageal reflux disease, gastric ulcer, gastritis, and duodenitis

In addition, from the guidelines for prevention and treatment of osteoporosis, 6 medical conditions (COPD, chronic kidney disease, thyrotoxicosis, hypofunction and other disorders of the pituitary gland, primary hyperparathyroidism, and Cushing’s syndrome) were included. COPD was most prevalent among those 6 medical conditions but less common compared to the 12 medical conditions. Prevalence rates of 4 out of the 6 medical conditions (thyrotoxicosis, hypofunction and other disorders of the pituitary gland, primary hyperparathyroidism, and Cushing’s syndrome) were very low among studied patients (less than 2%).

### Cost drivers for the fracture treatment cost

The result of regression analysis seeking drivers for the fracture treatment cost is presented in Table 4. Among demographic factors, both gender and age were significantly associated with the fracture treatment costs. Female had 1.08 times higher fracture treatment costs (95% CI 1.00 to 1.16) compared with male, and those aged 60–69, 70–79, and 80–89 had higher fracture treatment costs compared with those aged 50–59 (60–69: 1.20 [95% CI 1.01 to 1.42], 70–79: 1.26 [95% CI 1.07 to 1.48], and 80–89: 1.26 [95% CI 1.07 to 1.48]) after adjusting for other covariates (treatment duration [in month], fracture category [hip, vertebral, and NHNV], and comorbidities). In terms of treatment status among patients, a longer treatment period was associated with 1.05 times higher fracture treatment costs (95% CI 1.05 to 1.06). Non-hip fracture patients had significantly lower fracture treatment costs than hip fracture patients (vertebral 0.20 [95% CI 0.19 to 0.22] and NHNV 0.26 [95% CI 0.24 to 0.28]).

**Table 4:** Cost drivers for the fracture treatment cost by multivariable regression analysis ^a^

	Coefficients	95% CI Lower	95% CI Upper	*P* value
Gender (base = male)	1.08	1.00	1.16	< 0.05
Age (base = 50–59)				
60–69	1.20	1.01	1.42	< 0.05
70–79	1.26	1.07	1.48	< 0.01
80–89	1.26	1.07	1.48	< 0.01
90-	1.12	0.93	1.35	
Treatment period (month)	1.05	1.05	1.06	< 0.01
Fracture category (base = hip)				
Vertebral	0.20	0.19	0.22	< 0.01
NHNV	0.26	0.24	0.28	< 0.01
Comorbidities				
GI diseases	1.01	0.95	1.08	0.66
Hypertension	1.13	1.06	1.20	< 0.01
Other functional intestinal disorder	1.03	0.97	1.09	0.40
Dorsalgia	0.90	0.85	0.96	< 0.01
Disorders of lipoprotein metabolism and other lipidemia	1.00	0.94	1.06	0.98
Sleep disorders	1.03	0.97	1.10	0.29
Diabetes	0.97	0.91	1.04	0.38
Osteoarthritis of knee	1.01	0.94	1.07	0.83
Angina pectoris	1.05	0.98	1.13	0.20
Heart failure	0.95	0.88	1.03	0.19
Disorder of refraction and accommodation	0.86	0.80	0.93	< 0.01
Iron deficiency anemia	1.15	1.07	1.25	< 0.01
COPD	0.91	0.84	1.00	< 0.05
Chronic kidney disease	0.93	0.82	1.05	0.24
Thyrotoxicosis	1.11	0.91	1.39	0.32
Hypofunction and other disorders of the pituitary gland	1.09	0.68	1.94	0.73
Primary hyperparathyroidism	2.08	0.88	6.81	0.15
Cushing’s syndrome	0.46	0.14	3.27	0.30

^a^*n* = 12,898

Among comorbidities we identified, associations with the increased fracture treatment costs were suggested for hypertension and iron deficiency anemia (hypertension 1.13 [95% CI 1.06 to 1.20] and iron deficiency anemia 1.15 [95% CI 1.07 to 1.25]). On the contrary, associations with the decreased fracture treatment costs were observed for dorsalgia (0.90 [95% CI 0.85 to 0.96]), disorder of refraction and accommodation (0.86 [95% CI 0.80 to 0.93]), and COPD (0.91 [95% CI 0.84 to 1.00]).

An association was further investigated after limiting the type of patients to hip fracture and vertebral fracture patients (Tables 5). With regard to demographic factors, only the gender and the age of 70–79 years old retained association with the increased fracture treatment costs among hip fracture patients. An association with the treatment period was observed in both hip and vertebral fractures. Among comorbidities driving the fracture treatment cost in Table 4, only iron deficiency anemia was associated with increased fracture treatment costs in both hip and vertebral fractures. Associations between comorbidities and change in the fracture treatment costs other than disorder of refraction and accommodation were observed only in the hip fracture patient group. In addition, associations between the fracture treatment costs and two comorbidities were newly suggested among the hip fracture patient group. Sleep disorders were associated with the increased fracture treatment costs (1.11 [95% CI 1.04 to 1.19]), whereas diabetes was associated with the decreased fracture treatment costs (0.92 [95% CI 0.86 to 0.99]).

**Table 5:** Cost drivers for the fracture treatment cost in hip and vertebral fracture population by multivariable regression analysis

	Hip fracture patients (*n* = 2415)				Vertebral fracture patients (*n* = 6711)			
	Coefficient	95% CI Lower	95% CI Upper	*P* value	Coefficient	95% CI Lower	95% CI Upper	*P* value
Gender (base = male)	1.20	1.09	1.31	< 0.01	0.97	0.87	1.07	0.55
Age (base = 50–59)								
60–69	1.24	0.99	1.54	0.05	0.98	0.71	1.33	0.90
70–79	1.30	1.05	1.58	< 0.05	1.09	0.80	1.45	0.56
80–89	1.17	0.95	1.43	0.12	1.13	0.83	1.51	0.41
90-	0.99	0.79	1.22	0.91	1.09	0.77	1.53	0.62
Treatment period (month)	1.01	1.01	1.02	< 0.01	1.07	1.06	1.08	< 0.01
Comorbidities								
GI diseases	1.00	0.93	1.07	0.93	1.02	0.93	1.13	0.64
Hypertension	1.11	1.04	1.20	<0.01	1.04	0.95	1.15	0.39
Other functional intestinal disorder	1.02	0.95	1.09	0.60	1.03	0.94	1.13	0.49
Dorsalgia	0.93	0.86	1.00	<0.05	0.93	0.85	1.01	0.08
Disorders of lipoprotein metabolism and other lipidemia	1.07	0.99	1.15	0.07	1.05	0.95	1.16	0.34
Sleep disorders	1.11	1.04	1.19	< 0.01	1.01	0.91	1.11	0.89
Diabetes	0.92	0.86	0.99	< 0.05	0.95	0.86	1.06	0.33
Osteoarthritis of knee	1.05	0.97	1.14	0.24	1.11	1.00	1.23	0.06
Angina pectoris	1.08	1.00	1.17	0.06	1.06	0.94	1.20	0.33
Heart failure	0.96	0.89	1.05	0.37	0.94	0.83	1.07	0.35
Disorder of refraction and accommodation	0.99	0.90	1.09	0.83	0.82	0.74	0.92	< 0.01
Iron deficiency anemia	1.11	1.03	1.20	< 0.01	1.19	1.04	1.36	< 0.05
COPD	0.89	0.80	0.98	< 0.05	0.93	0.82	1.06	0.26
Chronic kidney disease	0.95	0.84	1.08	0.44	0.95	0.77	1.20	0.68
Thyrotoxicosis	1.03	0.80	1.37	0.82	1.24	0.89	1.79	0.23
Hypofunction and other disorders of the pituitary gland	1.18	0.67	2.35	0.59	0.95	0.46	2.41	0.90
Primary hyperparathyroidism	1.84	0.74	6.67	0.26	0.84	0.09	690.89	0.92
Cushing’s syndrome	0.45	0.13	3.24	0.29	0.48	0.08	22.56	0.55

### Cost drivers for the total medical cost in hip and vertebral fractures

Drivers for the total medical costs were also explored in the hip and vertebral fracture patient groups (Table 6). It was found that the total medical costs of female vertebral fracture were less than male vertebral fracture patients (−$655 [95% CI −$1018 to − 292]). The association between age and the increased total medical costs was observed only in vertebral fracture patients (aged 80–89: $1084 [95% CI $47 to $2120] and aged 90-: $1460 [95% CI $267 to $2652]).

**Table 6:** Cost drivers for the total medical cost in hip and vertebral fracture population by multivariable regression analysis

	Hip fracture patients (*n* = 2415)				Vertebral fracture patients (*n* = 6711)			
	Coefficients	95% CI Lower	95% CI Upper	*P* value	Coefficients	95% CI Lower	95% CI Upper	*P* value
Gender (base = male)	15	− 1146	1176	0.98	− 655	− 1018	− 292	< 0.01
Age (base = 50–59)								
60–69	42	− 2770	2853	0.98	332	− 748	1412	0.55
70–79	49	− 2551	2649	0.97	542	− 490	1575	0.30
80–89	746	− 1832	3324	0.57	1084	47	2120	< 0.05
90-	153	− 2593	2898	0.91	1460	267	2652	< 0.05
Treatment period (month)	194	95	293	< 0.01	− 2	− 38	33	0.90
1 year cost before fracture	− 0.003	− 0.004	− 0.003	< 0.01	− 0.001	− 0.002	− 0.001	< 0.01
Comorbidities								
GI diseases	174	− 732	1081	0.71	49	− 289	386	0.78
Hypertension	1972	1065	2879	< 0.01	1306	963	1649	< 0.01
Other functional intestinal disorder	1644	798	2490	< 0.01	1436	1114	1758	< 0.01
Dorsalgia	169	− 777	1115	0.73	− 426	− 724	− 127	< 0.01
Disorders of lipoprotein metabolism and other lipidemia	581	− 340	1501	0.22	− 34	− 387	318	0.85
Sleep disorders	1444	585	2302	< 0.01	474	133	815	< 0.01
Diabetes	557	− 368	1482	0.24	− 5	− 366	356	0.98
Osteoarthritis of knee	551	− 478	1580	0.29	− 278	− 640	85	0.13
Angina pectoris	358	− 670	1387	0.49	− 157	− 582	268	0.47
Heart failure	− 131	− 1171	909	0.81	386	− 43	815	0.08
Disorder of refraction and accommodation	773	− 431	1977	0.21	− 320	− 708	68	0.11
Iron deficiency anemia	2126	1153	3099	< 0.01	− 12	− 497	473	0.96
COPD	− 898	− 2210	415	0.18	58	− 389	505	0.80
Chronic kidney disease	4196	2441	5950	< 0.01	1285	483	2086	< 0.01
Thyrotoxicosis	1081	− 2400	4562	0.54	745	− 455	1944	0.22
Hypofunction and other disorders of the pituitary gland	2426	− 5459	10,312	0.55	− 171	− 2989	2646	0.91
Primary hyperparathyroidism	5341	− 8285	18,966	0.44	− 3127	− 14,867	8612	0.60
Cushing’s syndrome	− 9072	− 28,286	10,141	0.35	5036	− 3419	13,490	0.24

In terms of comorbidities, associations between the increased total medical costs and three medical conditions (hypertension, sleep disorders, and iron deficiency anemia) were observed as well (hypertension: $1972 [95% CI $1065 to $2879] for hip and $1306 [95% CI: $963 to $1649] for vertebral fracture, sleep disorders: $1444 [95% CI: $585 to $2302] for hip and $474 [95% CI: $133 to $815] for vertebral fracture, and iron deficiency anemia: $2126 [95% CI: $1153 to $3099] for hip). In contrast to the previous analysis, an association between dorsalgia and the decreased total medical costs was observed only among the vertebral fracture patient group (−$426 [95% CI: −$724 to −$127]). In this analysis, associations between two new comorbidities (other functional intestinal disorder and chronic kidney disease) and the increased total medical costs were observed (other functional intestinal disease: $1644 [95% CI: $798 to $2490] for hip, $1436 [95% CI: $1114 to $1758] for vertebral fracture, and chronic kidney disease: $4196 [95% CI $2441 to $5950] for hip, $1285 [95% CI: $483 to $2086] for vertebral fracture).

## Discussion

This study estimated the total medical costs and the fracture treatment costs in osteoporotic patients in Japan. We also explored the common comorbidities among osteoporotic fracture patients, and examined the potential cost drivers for the treatment costs.

Although a few studies have reported the treatment costs incurred for osteoporotic fracture in Japan, the number of patients was limited and the hospitals from which the patients were recruited was underrepresented. Our cost estimation was based on the latest claim data of 12,898 patients including both inpatients and outpatients, thus considered to be adequately representing Japanese population despite limitation of the database itself. Kondo et al. (2009) investigated the treatment costs of 41 hip fracture patients in three hospitals in Japan [13]. They reported that the costs were $19,034 on average. Although their result was slightly more expensive than what our study has found, it would be mainly due to the different payment system used by the hospital. The hospitals studied in this study were using only DPC payment system (similar to Diagnostic Related Group [DRG] in US) whereas the study by Kondo et al. included the hospitals with non-DPC payment system. DPC payment hospitals tend to manage their resource more effectively, and therefore it may lead to the lower treatment cost estimates. In contrast, Hagiwara et al. (2000) found that the mean treatment costs of hip fractures with 101 patients who were 60 years and older were 1,470,000 JPY ($13,364 when 1 USD = 110 JPY) [14]. This is less than what we have estimated, probably because their estimate was based on the inpatient stay in one hospital and did not include outpatient or rehabilitation care after discharge. Furthermore, it was reported that an average treatment costs for vertebral fracture was 780,000 JPY ($7091 when 1 USD = 110 JPY) with 272 hospitalized patients aged 60 years and older [15]. This can also be explained by the different study populations. Our study includes patients who were cared solely by outpatient visits; therefore, our research produced less expensive estimate than the research that only recruited hospitalized patients in terms of vertebral fracture patient costs.

The treatment costs borne by the patients with the second fracture were significantly higher compared with single fracture patient, which is consistent with previous studies in USA and UK [16–18]. In this study, those with the second vertebral fracture bore more than twice of total medical costs and fracture treatment costs when comparing their point estimates with those with a single vertebral fracture. This does not only suggest the importance of preventing the second fracture within 1 year from the index fracture but also indicate the great benefit of preventing the second fracture. From this perspective, prescription of osteoporosis drugs after the osteoporotic fracture can contribute in reducing financial burden of osteoporotic fracture.

The results of cost estimation indicate that total medical costs would be preferable in economic evaluation because the fracture treatment costs were almost only one third of total medical costs. Other two third would consist from non-fracture treatment costs such as doctor consultation costs, comorbidity treatment costs, and inpatient specific costs. The amount of these costs are not negligible, and hence they should be considered when evaluating the economic value of fracture treatment. To our best knowledge, there has been no study in Japan comparing the total medical costs and the fracture treatment costs, and the results would support the official guideline for the economic evaluation of drugs/medical devices recommending the inclusion of related comorbidity costs for cost parameters [19].

Interestingly, it was found that more fracture treatment costs were incurred among female hip fracture patients whereas less total medical cost was incurred female vertebral fracture patients, suggesting that more non-fracture (comorbidity) treatment costs were incurred among male than female. Male could be more likely to worsen their comorbidities. In fact, one study reported higher relative hazards of all-cause mortality after hip fracture in male during 0–1 year age interval [20]. Male may be more vulnerable after fractures, which lead to increasing non-fracture treatment cost.

Associations between comorbidities and treatment costs were explored, and nine comorbidities were detected as cost drivers. The associations with three medical conditions (hypertension, iron deficiency anemia, and diabetes) were consistent with previous research. Firstly, in our study, it was found that hypertension was associated with increased fracture treatment costs in hip fracture subgroups. This positive association was persistent when investigated in the total population. The association between hypertension and bone remodeling has been suggested [3], and one previous study showed that the BMD level of female patients with hypertension was lower than the BMD level of female patients without hypertension [21]. Another study further suggested that hypertension was a predictor for fracture nonunion [22]. Considering these empirical evidence, our result could additionally indicate that patients with hypertension have worse bone turnover, and it takes additional care to heal, leading to higher fracture treatment costs. This association was consistent with the total medical costs among hip and vertebral fracture patients.

Secondly, the result showed a strong positive association between iron deficiency anemia and treatment costs (both in the fracture treatment costs and the total medical costs among hip fracture patients). It was reported that several potential interactions between iron deficiency and bone loss (i.e., the role of iron in collagen synthesis or in vitamin D metabolism) [23]. Thus, it might be claimed that the patient with iron deficiency anemia bore more treatment costs due to its downside effect on bone metabolism. However, it should be noted that anemia can be an intermediate variable between other comorbidities and fractures as well. Anemia can be observed among the patients with chronic medical conditions such as CKD (the detail association between CKD and treatment costs will be explained later) [24]. Therefore, the estimated impact of iron deficiency anemia in this study may reflect the impact of such comorbidities on treatment costs.

Lastly, it is interesting to find that diabetes had an association with the decreased fracture treatment costs among hip fracture patients. One possible explanation for this negative association is the influence of some antidiabetic drugs. Although it has not been concluded yet, drugs such as insulin and metformin have had positive effects such as improved bone bridging or osteogenic effect in some experimental studies [25]. Therefore, patients with these antidiabetic drugs can have better bone quality and need less care. However, this should be further examined carefully as mixed results exist in this research area. For example, one recent study showed that the insulin use was negatively associated with bone mineral density increase [26]. In addition, this could be explained by behavioral aspect. One previous study in Japan which compared the healthcare resource use between osteoporosis diabetic patients and non-diabetic patients found that BMD testing was less frequently used among the patients with diabetes [27]. Although the reason behind this is unclear, our study results may indicate similar phenomenon.

Unpredicted positive associations were also found with three medical conditions (sleep disorders, CKD, and other functional intestinal diseases). Sleep disorders were positively associated with both the fracture treatment costs among hip fracture patients and the total medical costs among hip and vertebral fracture patients. Sleep disorders are common claims among the elderly population. In a previous study, it had potential associations not only with increasing risks of falls but also with osteoporosis [28], suggesting that sleep disorders may have some impact on bone metabolism. For example, Amstrap et al. (2015) showed that melatonin, the hormone regulating the sleep, improved BMD among women with osteoporosis [29]. Given this, our study may indicate that sleep disorders cause malignant bone metabolism among osteoporotic fracture patients, and consequently patients bear more total medical costs to recover from the fracture. Moreover, patients with CKD may also bear more total medical costs, although it was not observed in our analysis of fracture treatment costs. This may be because some osteoporosis treatment drugs such as bisphosphonates and painkillers can induce side effects such as hypocalcemia. Therefore, monitoring kidney conditions or treatments for such side effects may increase the total medical costs among CKD patients.

Lastly, other functional intestinal diseases had a positive association only with the total medical costs. Fracture patients would increase the risk of other functional intestinal disorder because of the physical inactivity during the hospitalization period. Vertebral compression fracture itself can cause constipation as complication [30]. Thus, it could be considered that those with other functional intestinal disorder represented the patient cohort with severe fractures and longer inpatient stay, which made their total medical costs higher than the costs of other patient group. Therefore, there may be no causal association between other functional intestinal disorder and treatment costs.

Our study also showed unexpected negative associations between treatment costs and the other three medical conditions (dorsalgia, disorder of refraction and accommodation, and COPD). Although the causal association between these medical conditions and treatment costs needs to be carefully interpreted with further research, potential causal pathways are hypothesized for each comorbidity as follows. The first one is dorsalgia. It had a negative association with the fracture treatment costs in hip fracture patients and the total medical costs in vertebral fracture patients, and the association between dorsalgia and fracture treatment costs remained in total population. The guidelines for prevention and treatment and osteoporosis suggest that dorsalgia is one of the factors for diagnosing osteoporosis [3]. General patients with dorsalgia during pre-fracture period might receive osteoporosis treatments earlier and therefore may not experience severe fractures. The second one is disorder of refraction and accommodation. It was negatively associated with the fracture treatment costs in vertebral fracture patients and total patient population, even after adjusting the demographic factors. One possible reason is that this variable may have worked as intermediate variable and reflected severity of other comorbidities such as diabetes, which is significant risk factor for retinopathy. The third one is COPD. It was demonstrated that COPD was associated with decrease in the fracture treatment costs among hip fracture patients. It was preserved even after the population was extended to total population. Given that the COPD is a well-known risk factor of osteoporosis and low BMD [3], this result looks counterintuitive. One potential association is that COPD patients often develop vertebral fractures, and therefore they could have already had preventive treatments before hip fracture, which would result in improving fracture recovery. However, further research is necessary.

The following limitations were recognized in this study. Firstly, a selection bias due to the nature of the database was possibly considered. The claim data used in this study were collected from acute care hospitals, allowing us to collect severer patients in Japan. On the other hand, these acute care hospitals may spend less treatment costs due to DRG-like payment system in Japan (DPC). In fact, it was shown that less treatment costs were incurred in DPC hospitals [13], which could underestimate total treatment costs. Furthermore, it could create the issue related to loss to follow-up. As there is no information regarding the clinical outcome of the patients, it is impossible to identify whether the patient was recovered, transferred to another care institution, or died. The Japanese government has introduced the policy that promotes specialization of medical institution into four categories (advanced acute-care, acute-care, rehabilitation-care, and long-term care) and collaboration between these hospitals in 2015 [31]. Hence, it is likely that the total medical costs of patients who were transferred to rehabilitation-care or long-term care institutions were not fully captured. In addition, accuracy of diagnosis record could be questioned. One study revealed that the validation indicators such as sensitivity or positive predictive value of diagnosis were varied depending on the medical conditions [32]. In this sense, generalizability of this study might be questioned as it is uncertain that the codes used in our study fully captured the osteoporotic fracture population. Finally, our study could not adjust patient behavioral and SES factors which could confound the results. Behavioral factors such as smoking and SES factor would not only affect patients’ health but also influence their treatment choice. These limitations should be considered carefully when interpreting the study results.

In conclusion, our study reported the treatment costs incurred to osteoporotic fracture patients in Japan and examined the cost drivers by analyzing the associations with comorbidities. To our knowledge, this is the first pragmatic study estimating the treatment costs of osteoporotic fracture patients through the analysis of large-sized RWD, and it showed financial burden of osteoporotic fracture in Japan including patients with the second fracture. This study also detected the novel associations between some comorbidities and the increase or decrease in treatment costs. The findings from this study would help clinicians, researchers, and policy makers understand the potential financial burden of osteoporotic fracture and would also support various health economic analyses in the future.
